# Spleen Tyrosine Kinase (SYK) is Necessary for cGAS‐STING Signaling in Müller Glia and Visual Function Deficits in Diabetic Mice

**DOI:** 10.1002/glia.70155

**Published:** 2026-04-13

**Authors:** Esma I. Yerlikaya, Siddharth Sunilkumar, Sandeep M. Subrahmanian, Allyson L. Toro, Clay T. Yeager, Kashif A. Shaikh, Edward W. Harhaj, Alistair J. Barber, Michael D. Dennis

**Affiliations:** ^1^ Department of Cell and Biological Systems Penn State College of Medicine Hershey Pennsylvania USA; ^2^ Department of Ophthalmology Penn State College of Medicine Hershey Pennsylvania USA

**Keywords:** chronic inflammation, diabetes, innate immunity, Müller glial cells, spleen tyrosine kinase

## Abstract

It is well established that inflammation contributes to the ocular complications caused by diabetes; however, the specific molecular events that drive diabetes‐induced pro‐inflammatory signaling in the retina remain to be fully elucidated. This study investigated the role of Müller glial spleen tyrosine kinase (SYK) in diabetes‐induced retinal complications. Hyperglycemic culture conditions increased mitochondrial membrane permeability and cytosolic mitochondrial DNA content in human MIO‐M1 Müller cells and enhanced cyclic GMP–AMP synthase (cGAS)–stimulator of interferon genes (STING) signaling, nuclear factor‐κB (NF‐κB) activation, and inflammatory cytokine expression. STING inhibition reduced inflammatory cytokine expression in cells exposed to hyperglycemic conditions by acting downstream of the increase in cytosolic mitochondrial DNA levels. In cells exposed to either hyperglycemic conditions or the STING agonist diABZI, SYK signaling was necessary for cGAS‐STING pathway activation. Inhibition of SYK‐dependent cGAS‐STING signaling reduced the expression of inflammatory cytokines, including IL1β, CCL2, and CCL5, under hyperglycemic conditions. In the retina of diabetic mice, Müller glia‐specific SYK deletion reduced glial activation and attenuated inflammatory cytokine expression. Müller glia‐specific SYK deletion also prevented diabetes‐induced retinal thinning and visual function deficits in spatial frequency threshold and contrast sensitivity. The data support an essential role for Müller glial SYK in diabetes‐induced retinal inflammation and the development of functional deficits in vision.

## Graphical abstract

- Hyperglycemic conditions increased mitochondrial membrane permeability and inflammatory signaling in Müller cells via cGAS‐STING activation.
- SYK was necessary for cGAS–STING activation and increased cytokine expression in Müller cells.
- Müller glia‐specific SYK deletion reduced glial activation and attenuated inflammatory cytokine expression in the retina of diabetic mice.
- Müller glia‐specific SYK deletion prevented diabetes‐induced retinal thinning and visual function deficits.

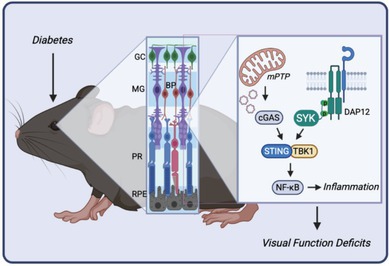

## 1 Introduction

Diabetic retinopathy (DR) is a common ocular complication caused by diabetes and the leading cause of vision loss. Current treatment options for DR almost exclusively focus on blocking the signaling of the pro‐angiogenic cytokine vascular endothelial growth factor (VEGF) and largely ignore changes in an array of pro‐inflammatory cytokines that have been implicated in retinal disease. Müller cells are the dominant retinal glial cell population and are well recognized for their role in producing homeostatic and trophic factors, including VEGF, that support the retinal vasculature, photoreceptors, and neurons (Reichenbach and Bringmann 2020). In response to diabetes, Müller cells become activated to support the survival of retinal neurons and combat retinal edema (Wang et al. 2010). However, with a prolonged duration of diabetes, Müller cells become gliotic and take on a maladaptive phenotype that contributes to defects in both neuronal and vascular cells of the retina (Coughlin et al. 2017; Yang et al. 2022). In response to diabetes, Müller glia undergo morphological and structural changes, including increased expression of glial fibrillary acidic protein (GFAP) and upregulated secretion of growth factors and cytokines that drive retinal complications (Shelton et al. 2009; Wang et al. 2010).

Examination of single‐cell RNA sequencing data from both diabetic patients and pre‐clinical models of diabetes supports robust upregulation of pro‐inflammatory signaling pathways that include the transcription factor nuclear factor κB (NF‐κB) specifically in retinal Müller glia (Zhang et al. 2022; Becker et al. 2021). NF‐κB promotes the expression of pro‐inflammatory factors, including interleukin 1 beta (IL‐1β), C‐C motif ligand 2 (CCL2/MCP‐1), CCL5/RANTES, and intercellular adhesion molecule 1 (ICAM1); and evidence supports the potential benefits of blocking NF‐κB activation in the context of DR (Zheng et al. 2007; Kowluru et al. 2003; Kanwar et al. 2007; Tuzcu et al. 2017; Ding et al. 2014). Recently, the cyclic GMP–AMP synthase (cGAS)–stimulator of interferon genes (STING) pathway has recently emerged as a critical regulator of NF‐κB signaling. While best known for its role in the innate immune response to microbial infection, activation of cGAS‐STING has also been reported in the development of sterile inflammation in the retina of patients with DR (Liu et al. 2023). In fact, STING was required for NF‐κB activation, enhanced inflammatory cytokine production, and capillary degeneration in the retina of diabetic mice (Liu et al. 2023). In addition to being activated by pathogen‐derived DNA, the cGAS‐STING pathway is initiated upon through mis‐localization of mitochondrial DNA (mtDNA) to the cytosol under stress conditions (West et al. 2015). When cytosolic mtDNA is detected by cGAS, it catalyzes the synthesis of the dinucleotide cyclic GMP‐AMP (cGAMP). Upon binding to cGAMP, STING undergoes several post‐translational modifications that promote its oligomerization and translocation from the ER to the ERGIC (ER‐Golgi Intermediate Compartment) and trans‐Golgi (Dobbs et al. 2015; Jeltema et al. 2023). At the Golgi, STING recruits Tank‐Binding Kinase 1 (TBK1), which phosphorylates STING at S366 and promotes the subsequent phosphorylation and activation of IRF3 and NF‐κB (Yum et al. 2021).

Phosphorylation of STING at Y240 by spleen tyrosine kinase (SYK) is an essential post‐translational modification required for STING activation (Wang et al. 2021). SYK is best known for its role in B cell development and activation (Mócsai et al. 2010); however, SYK has more recently been recognized for its roles in a variety of cells, including neurons, vascular endothelial cells, and glia (Yanagi et al. 2001). We recently discovered that in preclinical murine models of diabetes, signaling pathways leading to activation of SYK were upregulated in retinal Müller glia (Yerlikaya et al. 2022). The studies herein extend on the prior reports by investigating the role of diabetes‐induced SYK activation in Müller glial cGAS‐STING signaling and the inflammatory response of the retina in the context of diabetes.

## 2 Materials and Methods

### 2.1 Cell Culture

MIO‐M1 human Müller cells were incubated at 37°C and 5% CO_2_ in Dulbecco's Modified Eagle Medium (DMEM) containing 5.6 mM glucose supplemented with 10% inactivated fetal bovine serum (FBS) and 1% penicillin–streptomycin (P/S). Cells were exposed to culture medium supplemented with an additional 24.4 mM D‐Glucose or 24.4 mM mannitol as an osmotic control for 24 h. Lentiviral pLKO.1‐puro plasmids encoding shRNAs targeting either DAP12 or STING were obtained from the Penn State College of Medicine shRNA Library Core (RRID:SCR_021098). pLKO.1‐TRC was used as an shRNA control (Addgene Plasmid #10879). Lentivirus preparation, infection of MIO‐M1 cells, and clonal selection were performed as previously described (Yerlikaya et al. 2022). As previously tested and validated, shDAP12 was used to genetically manipulate SYK inhibition (Yerlikaya et al. 2022). Where indicated, culture medium was supplemented with 30 μM of SYK inhibitor ER‐27391 (Tocris Biosciences) or 1 μM of the STING inhibitor H‐151 (Invivogen) or STING agonist diABZI (Cayman Chemical). ER‐27391 was added to culture media 15 min prior to other manipulations. To evaluate NF‐κB activity, cells were cotransfected with NF‐κB‐TATA luciferase and pRL‐Renilla luciferase plasmids as previously described (Sunilkumar et al. 2022). Luciferase activity was measured using a Dual‐Luciferase Assay Kit (Promega). The Image‐iT LIVE Mitochondrial Pore Assay Kit was used to assess mitochondrial permeability transition pore (mPTP) opening.

### 2.2 Animals

At 6 weeks of age, diabetes was induced in male and female C57BL/6J SYK^fl/fl^ and Müller glia‐specific SYK knockout (GLAST‐CreER; SYK^fl/fl^, designated SYK mgKO) (RRID: IMSR_JAX: 012586; 017309) mice by low‐dose streptozotocin (STZ) administration. Male or female mice received 50 or 75 mg/kg STZ, respectively, by daily intraperitoneal (IP) injection for 5 consecutive days. Diabetes was confirmed by fasting blood glucose > 250 mg/dL. Two weeks after STZ administration, SYK deletion was induced by 4 daily IP injections of 30 mg/kg 4‐hydroxytamoxifen (4‐OHT). After 16 weeks of diabetes, mice were euthanized and whole eyes and retina isolates were collected for analysis. All procedures were performed in accordance with the ARVO statement on the ethical use of animals in ophthalmological research and were approved by the Penn State College of Medicine Institutional Animal Care and Use Committee (IACUC).

### 2.3 Western Blotting

Retinas were extracted, flash frozen in liquid nitrogen, and homogenized in 250 mL of extraction buffer as previously described (Dennis et al. 2015). Retinal extracts or cells were combined with Laemmli sample buffer and boiled for 5 min. Proteins were separated on a 4%–20% Criterion Protein Gel (Bio‐Rad) by SDS‐PAGE. Proteins were transferred to nitrocellulose membrane (Thermo Scientific) or PVDF membrane (Bio‐Rad) for western blotting. Membranes were blocked in 5% milk in TBST (50 mM Tris, pH 7.6; 0.9% NaCl; and 0.1% Tween‐20) and incubated overnight with the appropriate antibodies (Table S1).

### 2.4 Quantitative RT‐PCR

RNA was isolated from retinal tissue or cells using the TRIzol method or RNeasy Micro kit (Qiagen). cDNA was generated with the High‐Capacity cDNA Reverse Transcription Kit (ThermoFisher Scientific). qPCR was performed using the QuantiTect SYBR Green PCR Kit and the QuantStudio 12K Flex Real‐Time PCR System (RRID:SCR_021098). Results were normalized to GAPDH. Primer sequences are included in Table S2.

### 2.5 Measurement of Cytosolic Mitochondrial DNA

Cytosolic mitochondrial DNA was quantified using cell fractionation followed by qPCR, as previously described (Bronner and O'Riordan 2016). Cells were lysed with 0.1% NP‐40 and incubated on ice for 15 min, then centrifuged at 16,000 × *g* for 15 min at 4°C. Cytosolic mtDNA was isolated from the supernatant using the DNeasy Blood and Tissue Kit (Qiagen) per the manufacturer's protocol. qPCR was performed using the QuantStudio 12K Flex Real‐Time PCR System (Thermo Fisher Scientific, RRID:SCR_021098). Primer sequences for COXIII, CYTB are included in Table S2. Mean cycle threshold (Ct) values were obtained, and relative mRNA expression was calculated using 18S RNA as the internal control.

### 2.6 Immunofluorescence Microscopy

Whole eyes were collected and fixed in 4% paraformaldehyde (PFA, pH 7.5) for 30 min as previously described (Miller et al. 2022). Eyes were washed in PBS and incubated in 30% sucrose solution containing 0.05% sodium azide. Eyes were embedded in optimal cutting temperature compound, flash frozen, and sectioned. Ten μm cryosections were fixed in 2% PFA, permeabilized in PBS with 0.1% Triton X‐100, blocked with 10% normal donkey serum, and labeled with the appropriate antibodies (Table S1). Nuclei were counterstained with DAPI (1 μM) and slides were mounted using Fluoromount aqueous mounting media (Sigma). MIO‐M1 cells were seeded into 8‐well chamber slides (Corning). The following day, cells were exposed to hyperglycemic conditions or an osmotic control for 24 h. The cells were then permeabilized with 0.5% Triton X for 5 min, blocked with 3% BSA in PBS for at least 1 h at room temperature, and labeled with the appropriate antibodies (Table S1). Images were captured using an SP8 Confocal Laser Microscope (Leica Microsystems) with frame‐stack sequential scanning.

### 2.7 Histology and Morphometric Analysis

As described previously (Subrahmanian et al. 2025), 10 μm retinal cryosections were stained with H&E (Vector Laboratory) to assess retinal structural integrity and layer thickness. For thickness measurements, retinal cryosections were taken from approximately 100 μm lateral to the optic nerve head. Images of retinal sections were obtained and retinal thickness was measured using ImageJ software. Measurements were made at approximately 50 μm from the optic nerve to minimize positional variation within the retina. An observer masked to the sample identity obtained at least 5 measurements of retinal thickness per sample image.

### 2.8 Visual Function Testing

Behavioral optomotry was performed using an OptoMotry virtual optomotor system (Cerebral Mechanics), as previously described (Miller et al. 2016). Spatial frequency threshold and contrast sensitivity were assessed by monitoring the optomotor reflex elicited by a rotating vertical grating pattern projected by four inward‐facing screens. Spatial frequency threshold was assessed at 100% contrast. Contrast sensitivity was assessed at a spatial frequency of 0.092 cycles/degree. Spatial frequency threshold and contrast sensitivity were defined as the highest values to elicit reflexive head movement.

### 2.9 Statistical Analysis

Data are expressed as mean ± SD. Two‐way ANOVA was used to analyze data from experiments with more than two groups and pairwise comparisons were made using Tukey's test for multiple comparisons. Student *t*‐test was used to detect significant differences between two groups. Significance is indicated at a *p* value < 0.05 for all analyses. For immunofluorescence, colocalization between two channels (Calnexin and STING) was quantified by subtracting the background using the rolling ball radius and then calculating the Mander's coefficient of each region of interest (ROI), where each ROI represents a cell. All samples in an experiment were analyzed with the same parameters.

## 3 Results

### 3.1 Hyperglycemic Conditions Activated Pro‐Inflammatory Signaling in Müller Cells

**FIGURE 1:**
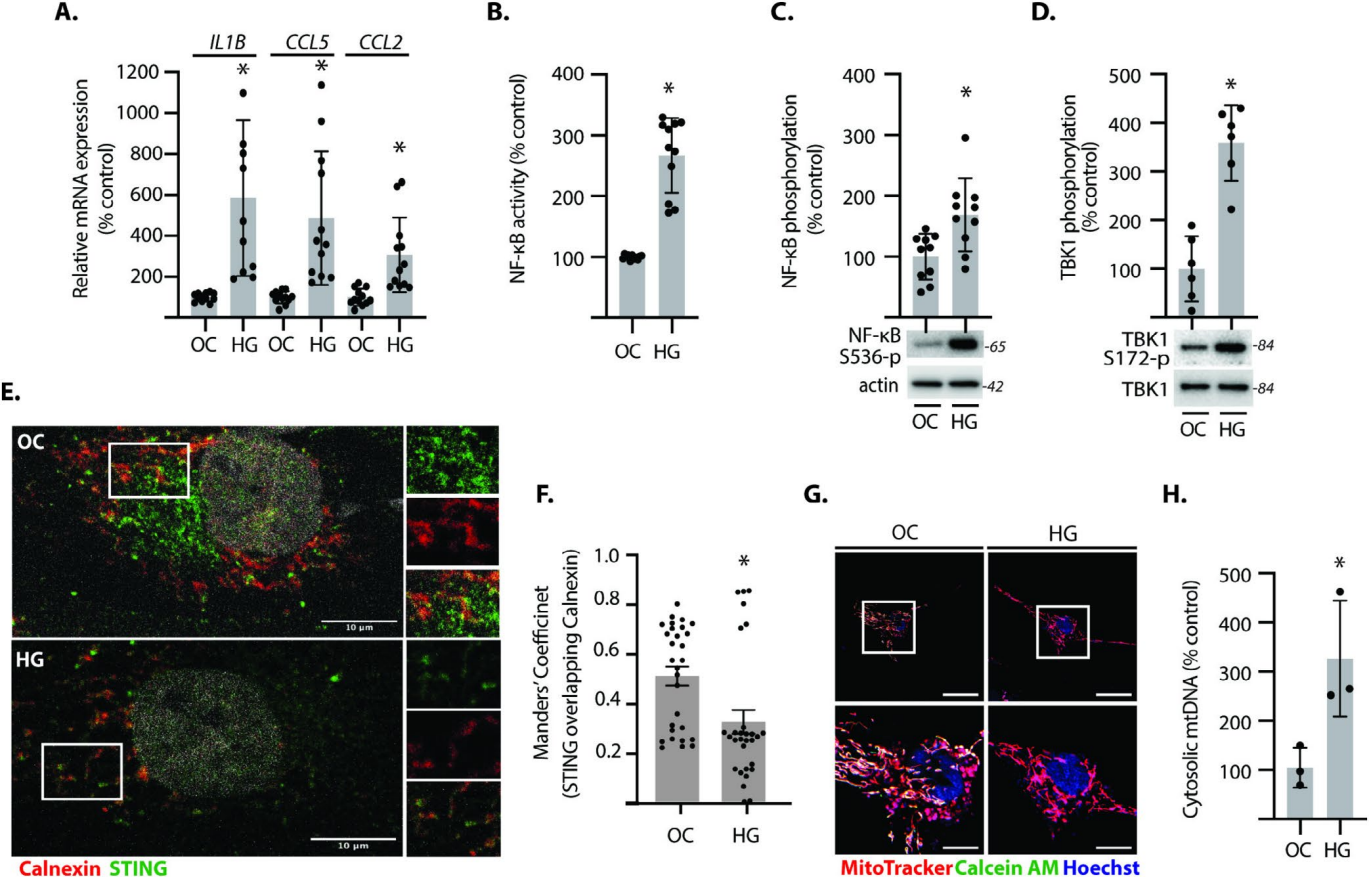
Hyperglycemic conditions promoted pro‐inflammatory signaling in Müller cells. Human MIO‐M1 Müller cells were cultured in media containing 5.6 mM glucose, followed by exposure to media containing either 30 mM glucose (HG) or an osmotic control (OC) containing 5.6 mM glucose plus 24.4 mM mannitol for 24 h. (A) *IL1B*, *CCL5*, and *CCL2* mRNA expression were determined in cell lysates by RT‐PCR. (B) NF‐κB activity was determined by dual luciferase assay. (C and D) Western blotting was used to evaluate phosphorylation of NF‐κB at S536 (C) and TBK1 at S172 (D) in cell lysates. Representative blots are shown. Molecular mass in kDa is indicated at the right of each blot. (E) STING and the ER marker Calnexin were visualized by immunofluorescence microscopy. (F) Colocalization of STING and Calnexin in (E) was quantified. (G) Mitochondrial permeability was evaluated by Calcein AM assay. MitoTracker was used to visualize mitochondria. Quenching of Calcein AM fluorescence by cobalt chloride reflects a compromised mitochondrial membrane. (H) Cytosolic mtDNA content was determined in fractionated cells by RT‐PCR. Values are means ± SD (*n* = 3–12). *p < 0.05 versus OC.

In Müller cells exposed to hyperglycemic conditions, the expression of mRNAs encoding the pro‐inflammatory factors IL1β, CCL5, and CCL2 was increased (Figure 1A). Hyperglycemic conditions also promoted NF‐κB activity (Figure 1B) and NF‐κB (Rel A) phosphorylation at S536 (Figure 1C). Consistent with cGAS‐STING pathway activation, TBK1 phosphorylation was enhanced in cells exposed to hyperglycemic conditions (Figure 1D). STING activation was also supported by the loss of its colocalization with the ER marker Calreticulin under hyperglycemic conditions (Figure 1E,F). Mitochondrial permeability was evaluated using a Calcein AM–cobalt chloride quench assay. In cells exposed to hyperglycemic conditions, Calcein AM fluorescence was lost, indicating mPTP opening (Figure 1G). Mitochondrial permeability was further evidenced by increased cytosolic mtDNA content upon exposure to hyperglycemic conditions (Figure 1H). Together, the data support a potential role for mtDNA leak and cGAS‐STING activation in the pro‐inflammatory response of Müller cells to hyperglycemic conditions.

### 3.2 STING Inhibition Prevented Pro‐Inflammatory Cytokine Expression in Müller Cells Exposed to Hyperglycemic Conditions

**FIGURE 2:**
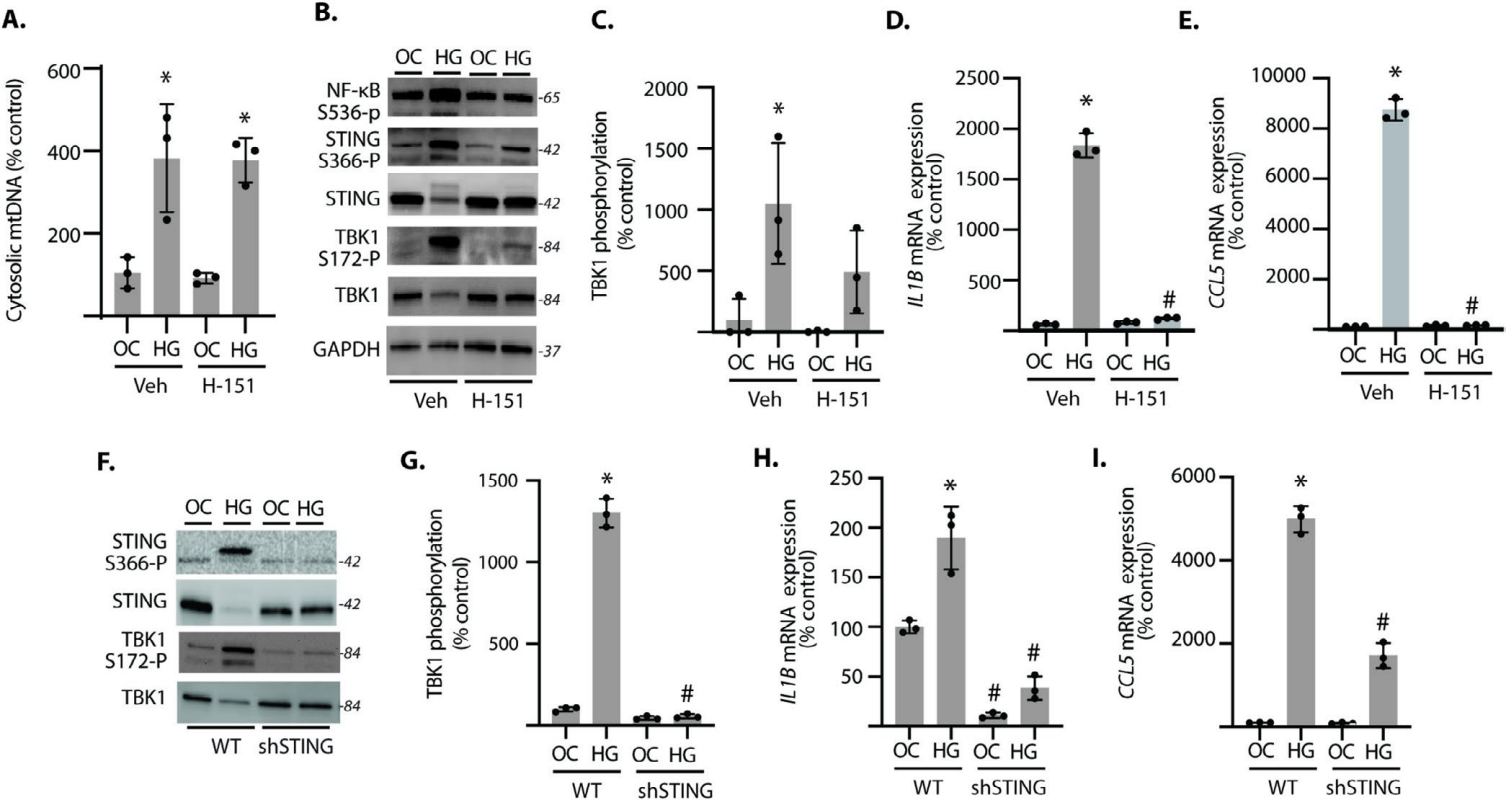
STING antagonism prevents increased pro‐inflammatory cytokine expression in Müller cells exposed to hyperglycemic conditions. MIO‐M1 Müller cells were cultured in media containing 5.6 mM glucose, followed by exposure to media containing either 30 mM glucose (HG) or an osmotic control (OC) containing 5.6 mM glucose plus 24.4 mM mannitol for 24 h. (A–E) Cells were exposed to the STING antagonist H‐151 or vehicle (Veh) for 12 h before OC or HG conditions. Cytosolic mtDNA content was determined in fractionated cells by RT‐PCR (A). Western blotting was used to evaluate phosphorylation of NF‐κB, STING, and TBK1 (B). TBK1 phosphorylation in (B) was quantified (C) *IL1B* and *CCL5* mRNA expression were determined in cell lysates by RT‐PCR (D and E). (F–I) STING was knocked down in MIO‐M1 cells by expression of a shRNA targeting the STING mRNA (shSTING). Western blotting was used to evaluate the phosphorylation of STING and TBK1 in wild‐type (WT) and shSTING cells (F). TBK1 phosphorylation in (F) was quantified (G) *IL1B* and *CCL5* mRNA expression were determined in cell lysates by RT‐PCR (H–I). Values are means ± SD (*n* = 3). *p < 0.05 versus OC. #*p* < 0.05 versus Veh or WT.

To evaluate the role of cGAS‐STING activation in the pro‐inflammatory response of Müller cells, STING was inhibited with the STING antagonist H‐151. STING antagonism did not prevent an increase in cytoplasmic mtDNA content in cells exposed to hyperglycemic conditions (Figure 2A). However, STING inhibition prevented the enhanced phosphorylation of NF‐κB, STING, and TBK1 in cells exposed to hyperglycemic conditions (Figure 2B,C). STING inhibition also prevented an increase in the expression of pro‐inflammatory factors in Müller cells exposed to hyperglycemic conditions (Figure 2D,E). Consistent with STING pharmacologic inhibition, STING knockdown by shRNA expression also inhibited TBK1 autophosphorylation (Figure 2F,G) and the expression of pro‐inflammatory factors (Figure 2H,I) in Müller cells exposed to hyperglycemic conditions. The data support a critical role for cGAS‐STING signaling in promoting inflammatory cytokine production under hyperglycemic conditions.

### 3.3 SYK Signaling Was Necessary for STING Activation in Response to Hyperglycemic Conditions

**FIGURE 3:**
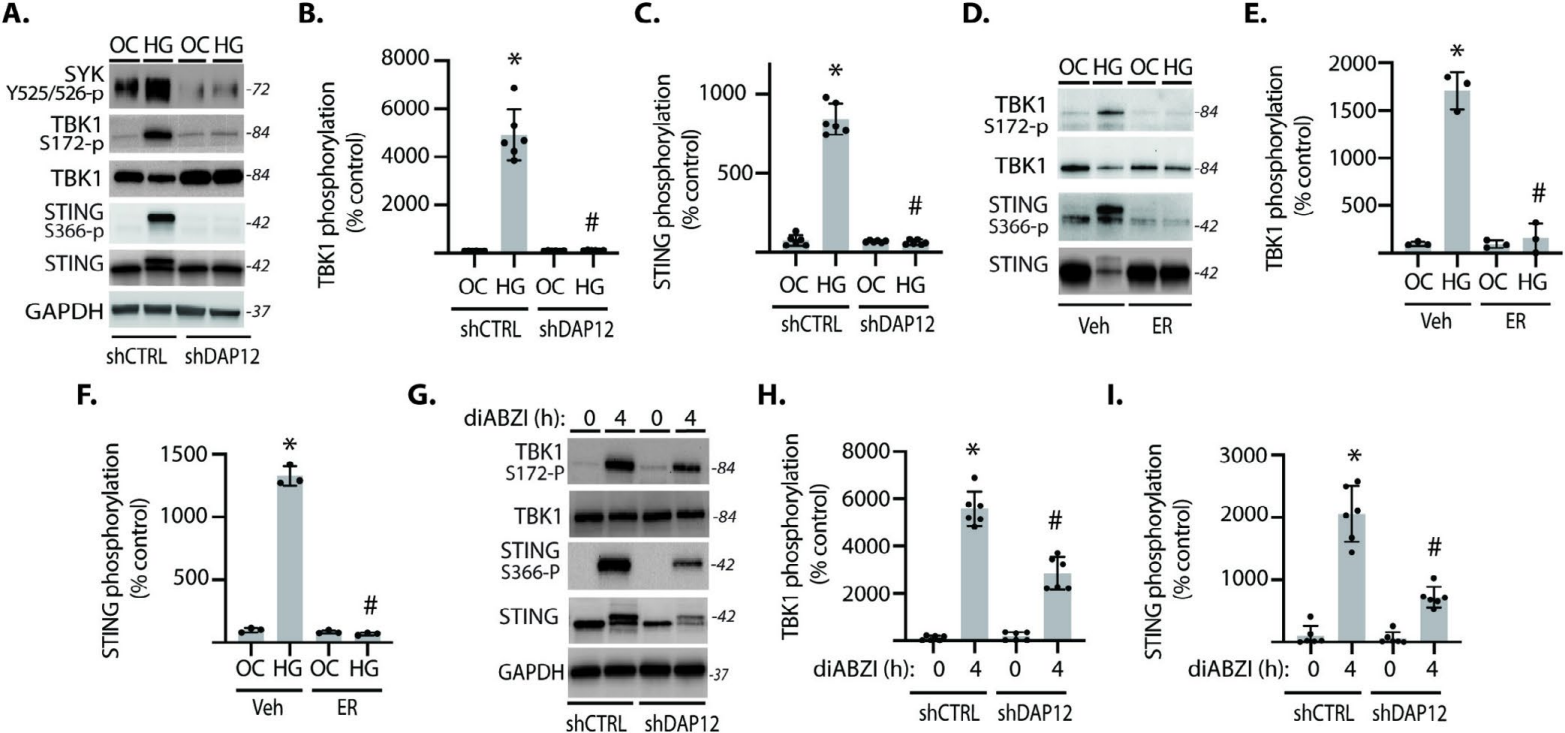
SYK signaling was necessary for cGAS‐STING pathway activation in Müller cells. (A–C) MIO‐M1 Müller cells were cultured in media containing 5.6 mM glucose, followed by exposure to media containing either 30 mM glucose (HG) or an osmotic control (OC) containing 5.6 mM glucose plus 24.4 mM mannitol for 24 h. DAP12 was knocked down by stable expression of a shRNA (shDAP12). Control cells expressed a control shRNA (shCTRL). SYK phosphorylation at Y525/526, TBK1 phosphorylation at S172, and STING phosphorylation at S366 were evaluated in cell lysates by western blotting. Representative blots are shown. Protein molecular mass in kDa is shown at right of blots. Quantification of TBK1 (B) and STING (C) phosphorylation in A is shown. (D–F) Cells were exposed to the SYK inhibitor ER‐27391 (ER) or a vehicle control (Veh). TBK1 and STING phosphorylation were evaluated in cell lysates by western blotting (D). Quantification of TBK1 and STING phosphorylation in D is shown in (E and F), respectively. (G–I) Cells were exposed to media supplemented with the STING agonist diABZI. Values are means ± SD (*n* = 3–6). *p < 0.05 versus OC or time 0. #*p* < 0.05 versus shCTRL or Veh.

To investigate a role for SYK in Müller glial cGAS‐STING signaling, DAP12 was knocked down by shRNA expression as previously described (Yerlikaya et al. 2022). SYK activation loop autophosphorylation at Y525/Y526 is a key marker of SYK kinase activity (Zhang et al. 2000). In control cells exposed to hyperglycemic conditions, SYK autophosphorylation was enhanced (Figure 3A). By contrast, DAP12 knockdown prevented an increase in SYK autophosphorylation upon exposure to hyperglycemic conditions. In association with the increase in SYK autophosphorylation, TBK1 autophosphorylation (Figure 3B) and STING phosphorylation (Figure 3C) were enhanced in cells exposed to hyperglycemic conditions. Importantly, DAP12 knockdown prevented the increase in TBK1 and STING phosphorylation. In support of the genetic manipulation, the SYK inhibitor ER‐27391 prevented an increase in TBK1 and STING phosphorylation in cells exposed to hyperglycemic conditions (Figure 3D–F). To further explore the impact of SYK on cGAS‐STING signaling, Müller cells were also exposed to the cGAMP mimetic diABZI (compound 3). In cells exposed to diABZI, TBK1 and STING phosphorylation were enhanced, and the effect was suppressed by DAP12 knockdown (Figure 3G–I).

### 3.4 SYK Was Necessary for Increased Pro‐Inflammatory Cytokine Expression in Cells Exposed to Hyperglycemic Conditions

**FIGURE 4:**
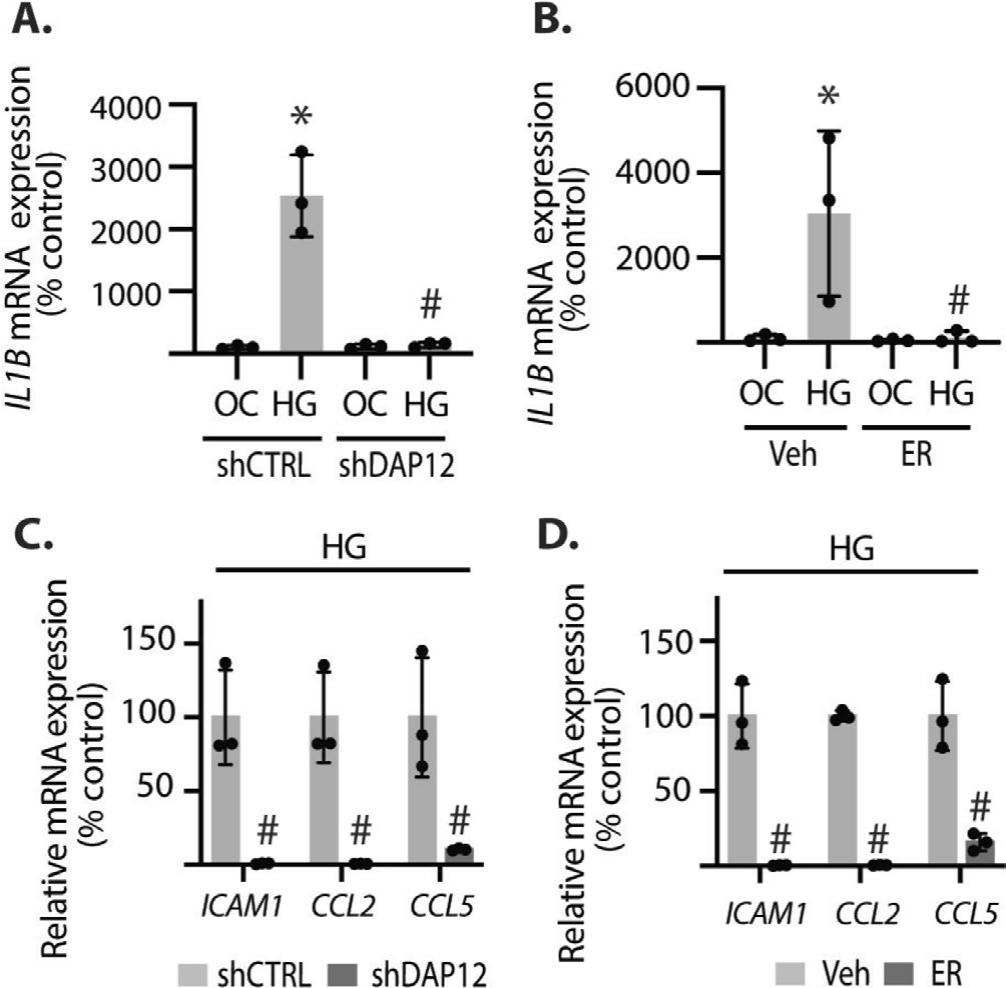
SYK inhibition suppressed pro‐inflammatory cytokine expression in Müller cells exposed to hyperglycemic conditions. MIO‐M1 Müller cells were cultured in media containing 5.6 mM glucose, followed by exposure to media containing either 30 mM glucose (HG) or an osmotic control (OC) containing 5.6 mM glucose plus 24.4 mM mannitol for 24 h. (A) DAP12 was knocked down by stable expression of a shRNA (shDAP12). Control cells expressed a control shRNA (shCTRL). *IL1B* mRNA expression was evaluated in cell lysates by RT‐PCR. (B) MIO‐M1 cells were exposed to the SYK inhibitor ER‐27391 (ER) or vehicle control (Veh) prior to exposure to HG/OC. (C and D) *ICAM*, *CCL2*, and *CCL5* were quantified in cells exposed to hyperglycemic conditions. Values are means ± SD (*n* = 3). *p < 0.05 versus time 0. #*p* < 0.05 versus shCTRL or Veh.

Based on the suppressive effect of SYK inhibition on cGAS‐STING activation, we also investigated the role of SYK signaling in the induction of pro‐inflammatory cytokines. DAP12 knockdown prevented an increase in *IL1B* expression in cells exposed to hyperglycemic conditions (Figure 4A). Moreover, there was a similar suppressive effect on *IL1B* expression in cells exposed to hyperglycemic conditions with SYK chemical inhibition (Figure 4B). DAP12 knockdown or ER‐27391 treatment also reduced the expression of *ICAM1*, *CCL2*, and *CCL5* under hyperglycemic conditions (Figure 4C,D).

### 3.5 Conditional Deletion of SYK in Retinal Müller Glia by Cre‐Lox Recombination

SYK and the Müller glia marker glutamine synthetase (GS) were visualized in retinal sections by immunofluorescence microscopy. SYK partially colocalized with GS (Figure S1A). More specifically, SYK colocalized with GS in both the cell bodies and end feet of Müller glia within the inner nuclear layer (INL) and ganglion cell layer (GCL), respectively. To specifically evaluate a role for Müller glial SYK in diabetes‐induced retinal defects, mice with Müller cell‐specific cre expression (JAX line 012586) were crossed with a strain that carries a floxed SYK allele (JAX line 017309) (Figure S1B). In the retina of SYK^fl/fl^ mice, SYK was visualized throughout the inner retina (Figure S1C). As compared to SYK^fl/fl^ mice, SYK abundance in the INL and GCL was reduced in SYK mgKO mice following 4‐OHT administration (Figure S1D).

### 3.6 Müller Glia‐Specific SYK Deletion Reduced Diabetes‐Induced Glial Activation and Inflammatory Cytokine Expression

**FIGURE 5:**
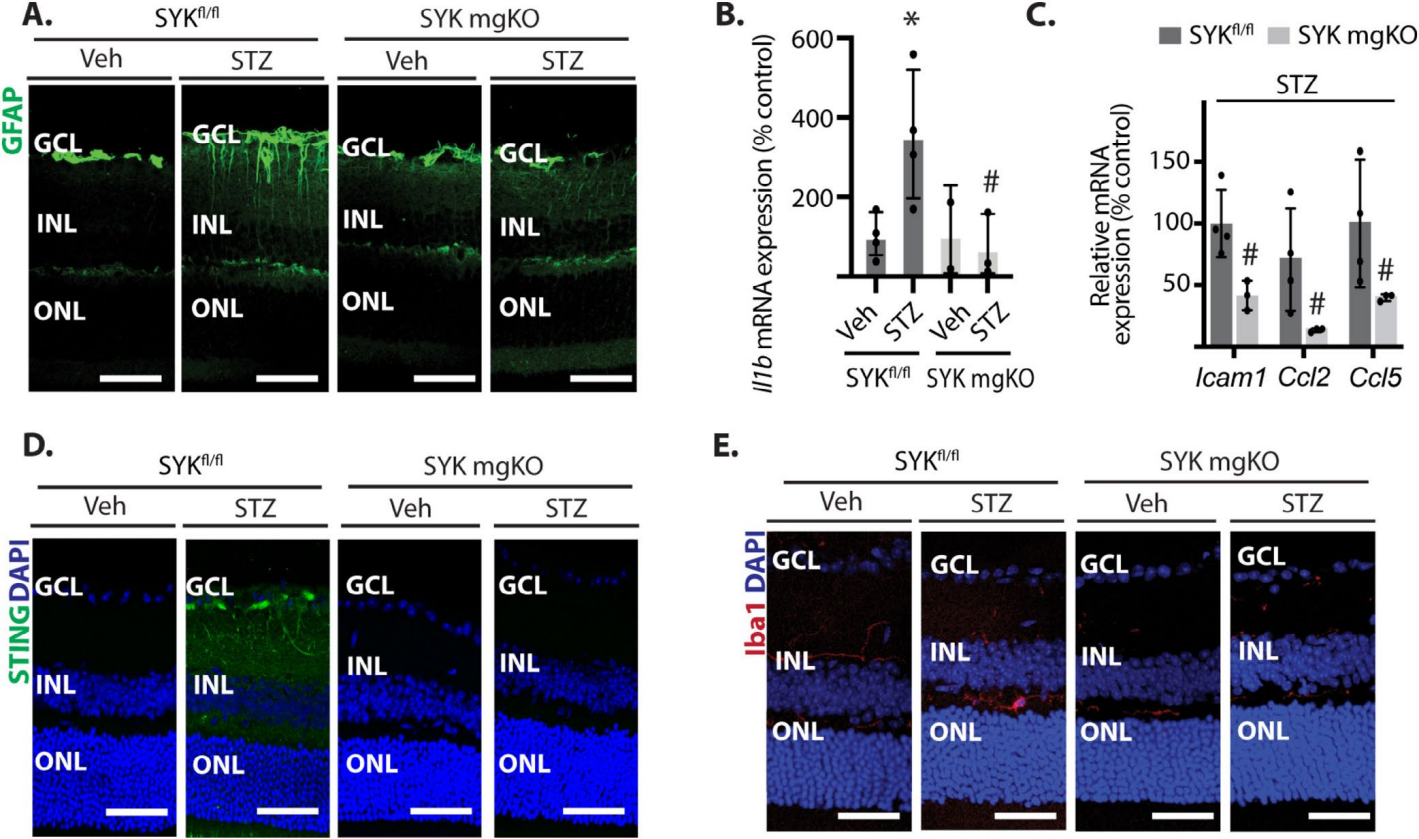
Müller glial SYK deletion reduced gliosis and inflammatory cytokine levels in diabetic mice. Diabetes and cre‐lox recombination were induced in SYK^fl/fl^ and SYK mgKO mice. (A) Glial fibrillary acidic protein (GFAP, green) was visualized in retinal sections by immunofluorescence. (B and C) *Il1b*, *Icam1*, *Ccl2*, and *Ccl5* mRNA expression were quantified in retinal lysates by RT‐PCR. (D) STING was visualized in retinal sections by immunofluorescence. Nuclei were counterstained with DAPI (*blue*). (E) Ionized calcium‐binding adaptor molecule 1 (Iba1, *red*) was visualized in retinal sections by immunofluorescence. Values are means ± SD (*n* = 3–4). *p < 0.05 versus Veh. #*p* < 0.05 versus SYK^fl/fl^. GCL, ganglion cell layer; INL, inner nuclear layer; ONL, outer nuclear layer.

Diabetes was induced in SYK^fl/fl^ and SYK mgKO mice by low‐dose streptozotocin administration (Figure S1E). Streptozotocin promoted a similar increase in fasting blood glucose concentrations in diabetic SYK^fl/fl^ and SYK mgKO mice (Figure S1F). Glial fibrillary acidic protein (GFAP) was visualized in the retina of diabetic mice as a marker for Müller cell gliosis. In the retina of diabetic SYK^fl/fl^ mice, GFAP immunoreactivity was enhanced (Figure 5A). Diabetes‐induced GFAP expression in the retina of SYK^fl/fl^ mice was localized to transverse processes, characteristic of Müller glia. Unlike diabetic wild‐type mice, Müller cell gliosis was absent in the retina of diabetic SYK mgKO mice. Expression of *Il1b* mRNA was also increased in the retina of diabetic mice in a manner that was dependent on Müller cell‐specific SYK expression (Figure 5B). Similar reductions in *Icam*1, *Ccl2*, and *Ccl5* mRNA expression were also observed in the retina of diabetic SYK mgKO mice compared to diabetic SYK^fl/fl^ mice (Figure 5C). Notably, STING protein levels were increased in the retina of diabetic SYK^fl/fl^ mice but remained low in diabetic SYK mgKO mice (Figure 5D). Diabetes also promoted microglial activation in the retina, as the number of Iba1‐positive cells with ameboid morphology was increased in SYK^fl/fl^ mice. By contrast, diabetic SYK mgKO mice showed no increase in microglial activation (Figure 5E).

### 3.7 Diabetes‐Induced Retinal Deficits Were Abolished by Müller Glia‐Specific SYK Deletion

**FIGURE 6:**
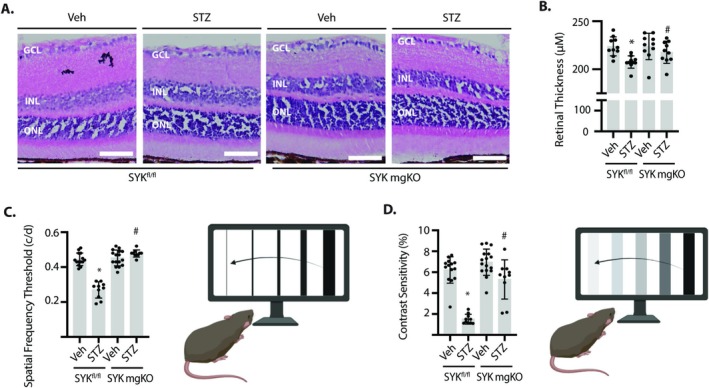
Diabetes‐induced retinal thinning and visual function deficits were prevented by Müller glia‐specific SYK deletion. Diabetes and cre‐lox recombination were induced in SYK^fl/fl^ and SYK mgKO mice. (A and B) H&E staining was used to visualize retinal structure. Retinal thickness in (A) was quantified in (B). (C and D) Visual function was evaluated by behavioral optometry, wherein a reflexive head movement is elicited by rotating bars of varying size or intensity. Spatial frequency threshold was evaluated at 100% contrast. Contrast sensitivity was determined at a spatial frequency of 0.092 cycles/degree. Thresholds were averaged over three trials on consecutive days. Contrast sensitivity is expressed as an inverse percentage. Values are means ± SD (*n* = 10–16). *p < 0.05 versus Veh. #*p* < 0.05 versus SYK^fl/fl^. Graphics were created with Biorender.com. GCL, ganglion cell layer; INL, inner nuclear layer; ONL, outer nuclear layer.

Retinal thickness was significantly decreased in diabetic SYK^fl/fl^ mice as compared to non‐diabetic controls (Figures 6A,B and S2). Retinal thickness was similar in non‐diabetic SYK^fl/fl^ and SYK mgKO mice, and there was no diabetes‐induced decrease in retinal thickness in SYK mgKO mice. To evaluate the impact of Müller glial SYK on visual function, the optomotor response was evaluated in mice by behavioral optometry. Diabetic SYK^fl/fl^ mice exhibited deficits in both spatial frequency threshold (Figure 6C) and contrast sensitivity (Figure 6D). Remarkably, both spatial frequency threshold and contrast sensitivity were greater in diabetic SYK mgKO mice as compared to diabetic SYK^fl/fl^ mice, such that there was no difference in either measure in diabetic SYK mgKO mice versus non‐diabetic SYK mgKO mice. The data are consistent with a role for Müller glial SYK in diabetes‐induced visual function deficits.

## 4 Discussion

**FIGURE 7:**
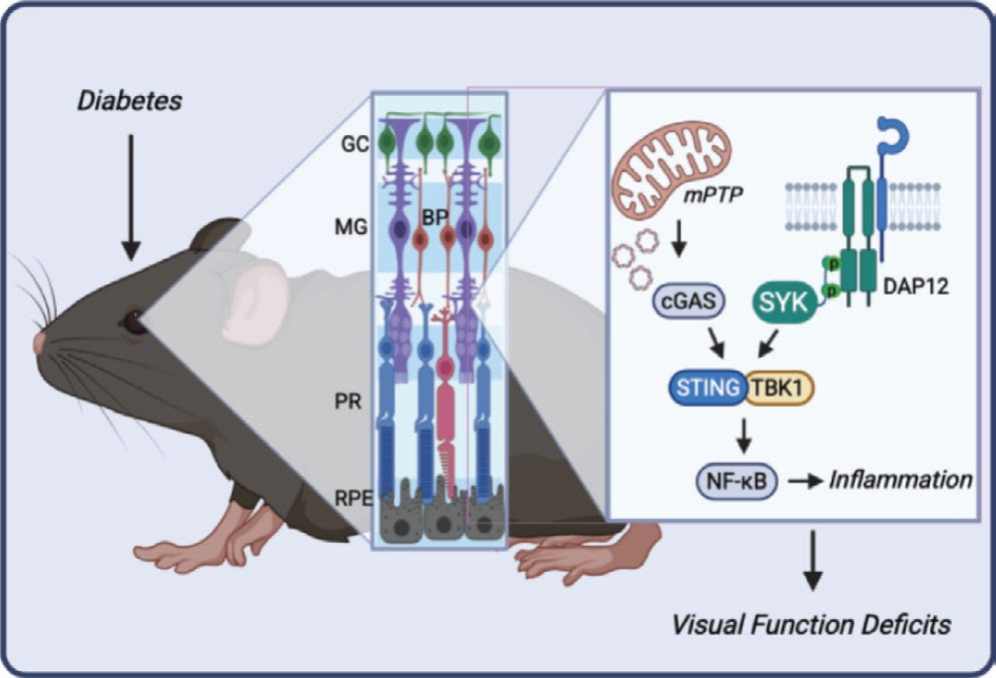
Müller glial SYK signaling contributes to diabetes‐induced retinal inflammation. Diabetes and hyperglycemic conditions activate retinal pro‐inflammatory signaling. Hyperglycemic conditions promote mitochondrial permeability transition pore (mPTP) opening and mtDNA leak. Cytoplasmic mtDNA activates the cGAS‐STING signaling pathway. SYK acts upstream of STING/TBK1 to enhance the activity of the transcription factor NF‐κB and the expression of pro‐inflammatory factors. Graphics were created with Biorender.com. BP, bipolar cells; GC, ganglion cells; MG, Müller glia; PR, photoreceptors; RPE, retinal pigmented epithelium.

Despite the central importance of Müller glia in DR pathology, there continues to be a significant knowledge gap regarding the precise mechanisms underlying their maladaptive response to diabetes. Herein, we investigated the role of SYK in promoting pro‐inflammatory signaling in retinal Müller glia. In human Müller cells exposed to hyperglycemic conditions, SYK autophosphorylation was enhanced. The observation is consistent with the increase in SYK activation loop autophosphorylation that is seen in the retina of murine models for both type 1 and type 2 diabetes (Yerlikaya et al. 2022). Genetic or pharmacologic SYK suppression inhibited pro‐inflammatory signaling in Müller cells, as SYK activity was necessary for activation of the cGAS‐STING pathway in response to either hyperglycemic conditions or the STING agonist diABZI. Moreover, Müller glia‐specific SYK deletion prevented glial activation, decreased retinal inflammatory cytokine expression, and protected visual function deficits in diabetic mice. Overall, the data support a working model in which diabetes promotes retinal pro‐inflammatory signaling by increasing mitochondrial permeability, mtDNA leak, and SYK‐dependent cGAS‐STING signaling in Müller glia (Figure 7).

Increased production of pro‐inflammatory factors contributes to the development and progression of DR (Rübsam et al. 2018). An array of inflammatory cytokines and chemokines is increased in the serum, vitreous, and aqueous humor of diabetic patients (Kern 2007). In diabetic patients without clinically visible signs of DR, expression of adhesion molecules such as ICAM‐1 is increased, which contributes to leukostasis (Vujosevic et al. 2015, 2016; Khalfaoui et al. 2008). Indeed, clinical and preclinical studies support the potential benefits of inhibiting pro‐inflammatory molecules to address diabetes‐induced retinal disease (Stahel et al. 2016; Yang et al. 2009). The expression of pro‐inflammatory cytokines is upregulated in the retina of diabetic rodents, and treatment with anti‐inflammatory agents is inhibitory toward the development of vascular lesions (Yang et al. 2009). In the retina of diabetic mice with Müller glia‐specific SYK deletion, the expression of *Icam1, Il1b, Ccl2*, and *Ccl5* was reduced as compared to control diabetic mice. SYK suppression also reduced the expression of these same pro‐inflammatory factors in Müller cell cultures exposed to hyperglycemic conditions. Together, the data are consistent with a role for SYK in diabetes‐induced retinal inflammation.

NF‐κB is a central mediator of pro‐inflammatory molecule expression, and aberrant NF‐κB signaling in response to diabetes and hyperglycemic conditions has been implicated in DR (Kanwar et al. 2007; Tuzcu et al. 2017; Ding et al. 2014; Liu et al. 2023; West et al. 2015). Indeed, germline disruption of STING reduces NF‐κB signaling and vascular pathology in the retina of STZ‐diabetic mice (Liu et al. 2023). The role of cGAS‐STING signaling in retinal disease is a rapidly growing area of interest that remains relatively underexplored in the context of DR. There are only a handful of studies that link cGAS‐STING activation to DR pathology, and they are almost exclusively focused on retinal endothelial cells (Liu et al. 2023; Guo et al. 2020). A recent study supports that signaling via the farnesoid X nuclear receptor and cGAS‐STING contributes to Müller glial activation in response to diabetic conditions (Wang et al. 2025). In support of a role for diabetes‐induced cGAS‐STING signaling in Müller glia, sequencing analysis of ribosome‐associated mRNAs isolated from Müller glia identified *Interferon Signaling* as the Top Canonical Pathway altered by STZ‐diabetes (Yerlikaya et al. 2022).

In Müller cell cultures exposed to hyperglycemic conditions, NF‐κB phosphorylation at S536 was enhanced in a manner that required SYK activity. The observation supports a prior study demonstrating that SYK promotes NF‐κB activation in RAW264.7 macrophages exposed to lipopolysaccharide (LPS) (Wang et al. 2021). In that study, SYK acted upstream of the inhibitor of κB (IκB) to promote NF‐κB activity in response to LPS (Wang et al. 2021). Canonical NF‐κB signaling is controlled by IκB, which acts to sequester NF‐κB in the cytoplasm. IκB kinase (IKK) phosphorylates IκB to promote its proteasomal degradation, IKK‐dependent phosphorylation of NF‐κB at S536, and ultimately NF‐κB nuclear translocation. In the retina of STZ‐diabetic mice, NF‐κB activity is increased concomitant with IKK activation, reduced IκB protein abundance, and enhanced NF‐κB phosphorylation at S536 (Sunilkumar et al. 2022). The observation is consistent with a model wherein diabetes acts upstream of IKK to promote NF‐κB activation and inflammatory cytokine production. The cGAS‐STING signaling pathway promotes NF‐κB activity by acting upstream of IKK. More specifically, STING‐induced NF‐κB signaling is elicited by TBK1/IKKε, which act independently of Toll‐like receptors (TLRs) to activate IKK (Balka et al. 2020). The data here support that increased NF‐κB signaling in Müller cells exposed to hyperglycemic conditions requires SYK‐dependent activation of cGAS‐STING signaling.

This study extends on two prior reports demonstrating the beneficial effects of SYK suppression on retinal pathology in diabetic rodents (Su et al. 2020; Liu et al. 2021). Oral administration of the SYK inhibitor R406 to diabetic rats was previously found to reduce retinal vascular injury and blunt VEGF expression in retinal tissue (Su et al. 2020). Notably, systemic delivery of R406 normalized fasting blood glucose concentrations and body weights in rats administered STZ. Thus, the therapeutic benefits of oral R406 in preventing retinal complications were likely due to improvements in insulin action, as it essentially prevented the diabetes phenotype. However, conditional SYK deletion in microglia was found to reduce microglial activation and pro‐inflammatory cytokine expression in the retina of STZ‐diabetic mice (Liu et al. 2021). In support of SYK expression in multiple retinal cell types, the majority of retinal SYK expression remained after microglia‐specific deletion (Liu et al. 2021). Herein, SYK was found to partially localize to the cell bodies and end feet of Müller glia in murine retina, and Müller glia‐specific SYK deletion reduced retinal SYK content. Thus, SYK is expressed in both retinal microglia and macroglia and potentially mediates diabetes‐induced defects in retinal glia. In support of a critical role for Müller glia‐specific SYK signaling in diabetes‐induced retinal defects, glial activation, increased retinal inflammatory cytokine expression, and visual function in STZ‐diabetic SYK mgKO mice were similar to non‐diabetic control mice. The observation provides evidence that SYK signaling in retinal glia impacts other cells of the retina and potentially contributes to visual impairment. Thus, the studies here support SYK as a novel therapeutic target to treat DR.

## Author Contributions

E.I.Y., A.J.B., E.W.H., and M.D.D. conceptualization. E.I.Y. and M.D.D. methodology. E.I.Y. and M.D.D. formal analysis. E.I.Y., S.S., S.M.S., A.L.T., C.T.Y., and K.A.S. investigation. A.J.B, E.W.H., and M.D.D. resources. E.I.Y. and M.D.D. data curation. E.I.Y. and M.D.D. writing original draft. S.S., M.S.S., A.L.T., C.T.Y., K.A.S., E.W.H., A.J.B., and M.D.D. reviewing and editing. E.I.Y. and M.D.D. visualization. S.S, S.M.S, A.J.B, E.W.H., and M.D.D. supervision. E.I.Y. and M.D.D. funding acquisition. M.D.D. is the guarantor of this work and, as such, has full access to all the data in the study and takes responsibility for the integrity of the data and the accuracy of the data analysis.

## Funding

These studies were supported by National Institutes of Health Grants R01 EY029702 and R01 EY032879 (to M.D.D.) and F31 EY037121 (to E.I.Y.).

## Ethics Statement

All procedures were performed in accordance with the ARVO statement on the ethical use of animals in ophthalmological research and were approved by the Penn State College of Medicine Institutional Animal Care and Use Committee (IACUC).

## Conflicts of Interest

The authors declare no conflicts of interest.

## Supporting information


**Figure S1:** Conditional SYK deletion was achieved in retinal Müller glia by cre‐lox recombination. (A) Glutamine synthetase (GS, green) and SYK (red) were visualized in murine retinal sections by immunofluorescence. (B) PCR products from genotyping mouse ear punch samples showed the wild‐type SYK PCR product at 234 base pairs (bp) and the floxed SYK variant at 349 bp. The PCR band for GLAST‐Cre expression was observed at 600 bp with the internal reaction control at 300 bp. (C) Cre‐lox recombination was achieved by 4‐hydroxytamoxifen administration. SYK (red) was visualized in the retina of SYK^fl/fl^ and SYK mgKO mice. Nuclei were counterstained with DAPI (blue). (D) SYK staining in C was quantified using Image J. (E) Streptozotocin (STZ) was administered by intraperitoneal injection to induce diabetes. Non‐diabetic mice received a vehicle control (Veh). (F) Fasting blood glucose concentrations were determined after 16 weeks of diabetes. Values are means ± SD (*n* = 3–4). *p < 0.05 versus SYK^fl/fl^ or Veh. GCL, ganglion cell layer; INL, inner nuclear layer; ONL, outer nuclear layer.

**Figure S2:** Diabetes‐induced retinal thinning was prevented by Müller glial SYK deletion. Diabetes and cre‐lox recombination were induced in SYK^fl/fl^ and SYK mgKO mice. H&E staining was used to visualize retinal cryosections taken at approximately 100 μm lateral to the optic nerve head. For each section, retinal thickness was determined at 50 μm from the optic nerve (red line). Representative zoomed images (red box) and quantifications are shown in Figure 6A,B.

**Table S1:** Antibody list.

**Table S2:** PCR oligonucleotide sequence.

## Data Availability

Primary data including unedited western blot images are available at https://doi.org/10.6084/m9.figshare.29620988. Excel files with raw data supporting the findings are publicly available from Scholarsphere.
