# Supplementary figures and images for: Spleen Tyrosine Kinase (SYK) is Necessary for cGAS‐STING Signaling in Müller Glia and Visual Function Deficits in Diabetic Mice
Source: Glia. 2026 Apr 13;74(6):e70155. doi: 10.1002/glia.70155 (PMC13073074; doi:10.1002/glia.70155)

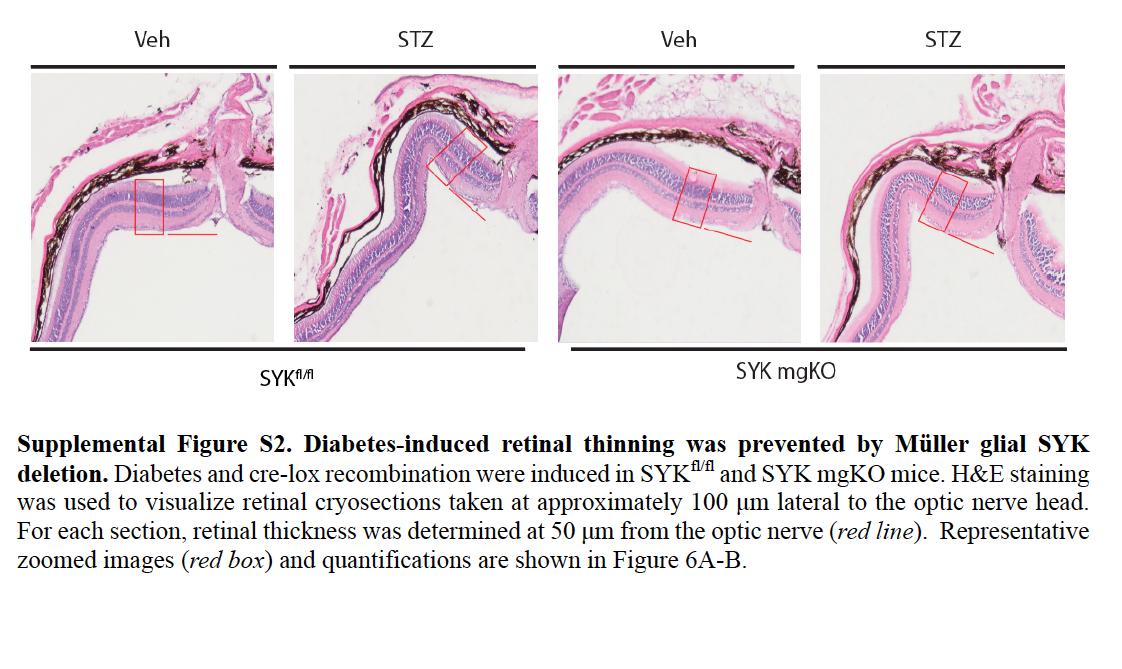

Supplement: Supplementary file 1 — Figure S1: Conditional SYK deletion was achieved in retinal Müller glia by cre‐lox recombination. (A) Glutamine synthetase (GS, green) and SYK (red) were visualized in murine retinal sections by immunofluorescence. (B) PCR products from genotyping mouse ear punch samples showed the wild‐type SYK PCR product at 234 base pairs (bp) and the floxed SYK variant at 349 bp. The PCR band for GLAST‐Cre expression was observed at 600 bp with the internal reaction control at 300 bp. (C) Cre‐lox recombination was achieved by 4‐hydroxytamoxifen administration. SYK (red) was visualized in the retina of SYKfl/fl and SYK mgKO mice. Nuclei were counterstained with DAPI (blue). (D) SYK staining in C was quantified using Image J. (E) Streptozotocin (STZ) was administered by intraperitoneal injection to induce diabetes. Non‐diabetic mice received a vehicle control (Veh). (F) Fasting blood glucose concentrations were determined after 16 weeks of diabetes. Values are means ± SD (n = 3–4). *p < 0.05 versus SYKfl/fl or Veh. GCL, ganglion cell layer; INL, inner nuclear layer; ONL, outer nuclear layer. [file GLIA-74-0-s004.png]

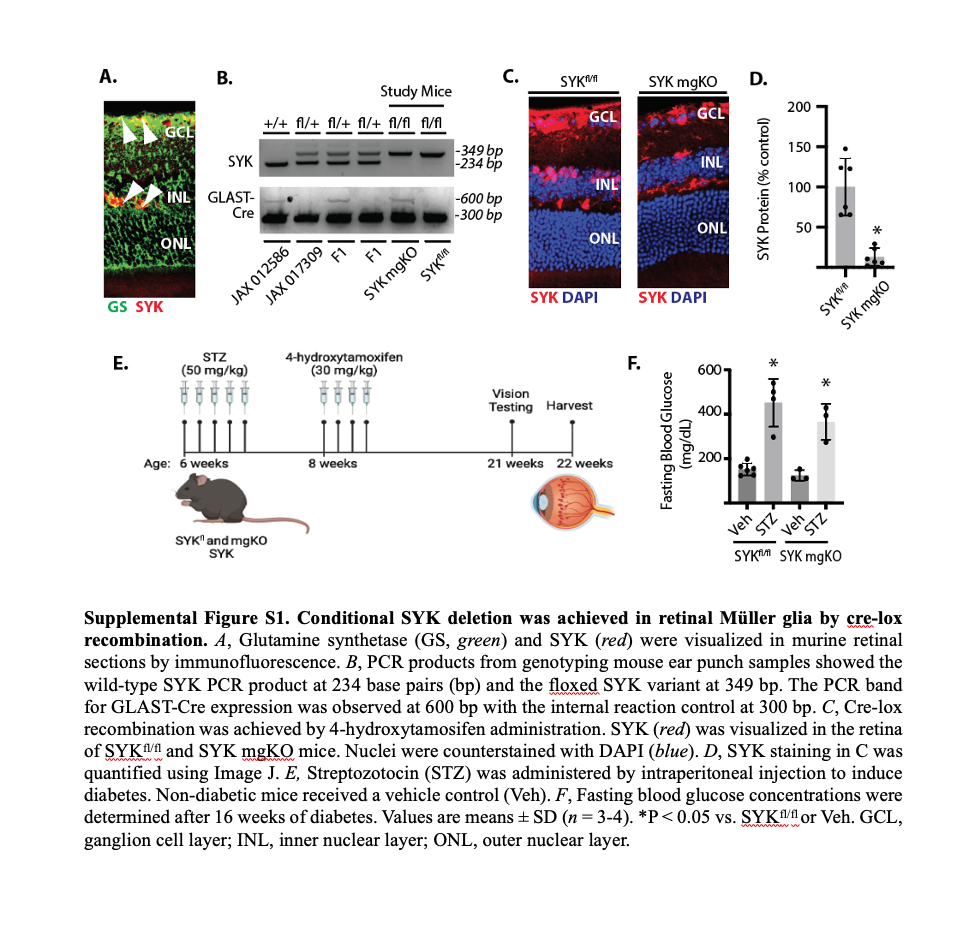

Supplement: Supplementary file 2 — Figure S2: Diabetes‐induced retinal thinning was prevented by Müller glial SYK deletion. Diabetes and cre‐lox recombination were induced in SYKfl/fl and SYK mgKO mice. H&E staining was used to visualize retinal cryosections taken at approximately 100 μm lateral to the optic nerve head. For each section, retinal thickness was determined at 50 μm from the optic nerve (red line). Representative zoomed images (red box) and quantifications are shown in Figure 6A,B. [file GLIA-74-0-s001.png]

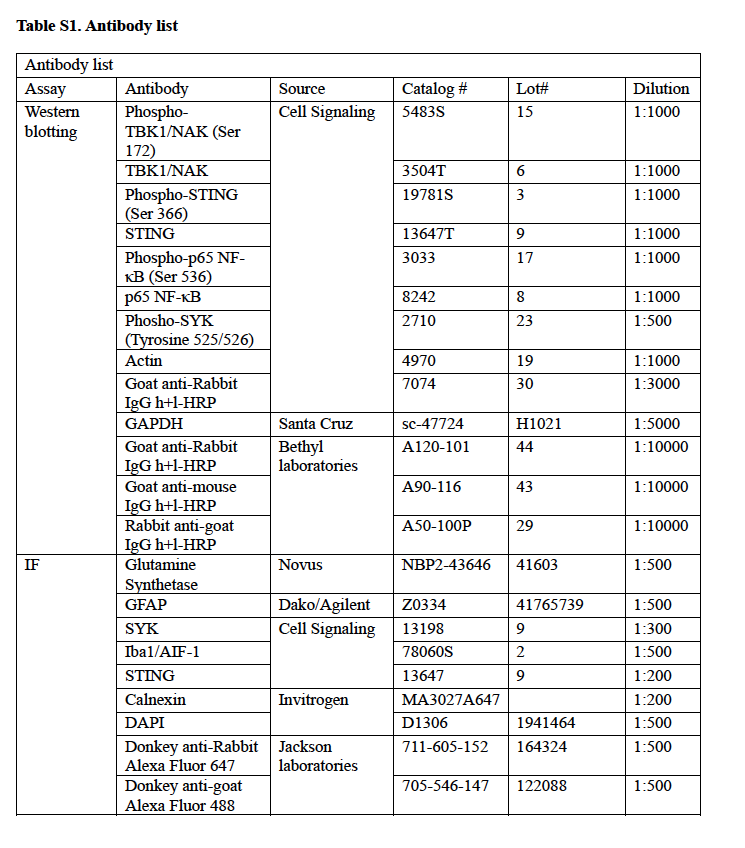

Supplement: Supplementary file 3 — Table S1: Antibody list. [file GLIA-74-0-s002.png]

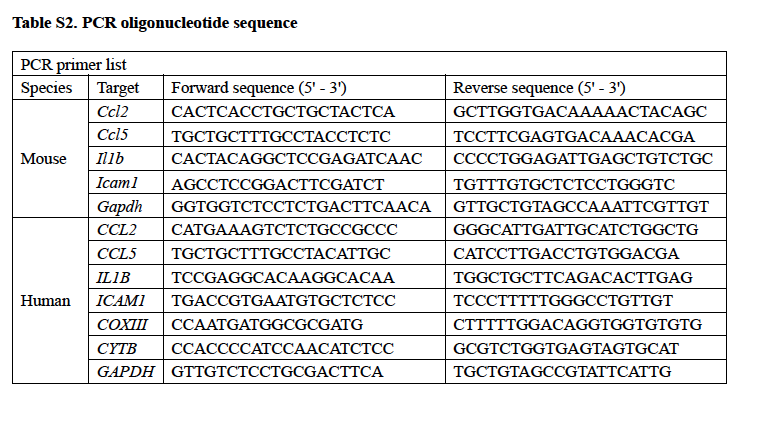

Supplement: Supplementary file 4 — Table S2: PCR oligonucleotide sequence. [file GLIA-74-0-s003.png]
